# Erchen Decoction Ameliorates Lipid Metabolism by the Regulation of the Protein CAV-1 and the Receptors VLDLR, LDLR, ABCA1, and SRB1 in a High-Fat Diet Rat Model

**DOI:** 10.1155/2018/5309490

**Published:** 2018-10-08

**Authors:** Shanshan Ding, Jie Kang, Ling Tong, Yuchen Lin, Linghong Liao, Bizhen Gao

**Affiliations:** ^1^College of Traditional Chinese Medicine, Fujian University of Traditional Chinese Medicine, Fuzhou 350122, China; ^2^Fujian Key Laboratory of TCM Health State, Fujian University of Traditional Chinese Medicine, Fuzhou 350122, China

## Abstract

Lipid metabolism disorder is a common metabolic disorder characterized by abnormal lipid levels in blood. Erchen decoction (ECD) is a traditional Chinese medicine prescription, which is used for the treatment of diseases caused by retention of phlegm dampness. It has been reported to ameliorate the disorder of lipid metabolism. The aim of the present study was to investigate the effects and underlying mechanisms of ECD in lipid metabolism disorder induced by a high-fat diet (HFD) in rats. ECD (4.35g/kg/d) and atorvastatin (10mg/kg/d, positive control) were orally administered to HFD-fed rats for four weeks. The parameters, food, water consumption, body weight, body length, liver, and visceral fat weight and the content of serum lipids and lipid transporters were assessed. The effects of ECD on the mRNA and protein expression levels of lipid transport factors were measured by real-time PCR and western blotting. The present study demonstrated that ECD improved the disorders of serum lipid and lipid transporters in HFD-fed rats, TG (0.70±0.08 mmol/L,* p*<0.01), LDL-C (1.50±0.19 mmol/L,* p*<0.01), LDL (1.38±0.21 mmol/L,* p*<0.05), and oxLDL (1.77±0.39 ng/mL,* p*<0.05) were downregulated, while HDL-C (0.87±0.13 mmol/L,* p*<0.01), FFA (0.62±0.13 mmol/L,* p*<0.05), HDL (38.8±4.0 mg/dL,* p*<0.05), and CETP (903.6±120.0 ng/mL,* p*<0.05) were upregulated. But ECD obviously had no effects on the indices food/water/energy intake, body/tissue (liver and fat) weight, and BMI (*p*>0.05). Concomitantly, ECD reversed the abnormal expressions of those lipid transport factors in the liver and visceral fat.

## 1. Introduction

Lipid metabolism disorder refers to a panel of diseases that are characterized by the disruption of lipid metabolism, such as LDL-hypercholesterolemia, hypertriglyceridemia, mixed hyperlipoproteinemia, and low HDL cholesterol [1]. These disorders are accompanied by abnormal lipid biochemical indices that are usually elevated and include exceeding serum levels of total cholesterol (TC), triglyceride (TG), low density lipoprotein cholesterol (LDL-C), and/or lower level of high density lipoprotein cholesterol (HDL-C) [2]. Lipid metabolism disorder is one of the major risk factors of fatty liver, diabetes, coronary heart disease, atherosclerosis, hypertension, and various cardiovascular diseases [3]. The LDL cholesterol has been recommended as the predominant marker for the early prognosis of cardiovascular diseases [1]. The current European recommendations state that an LDL-cholesterol target value is defined on the basis of the overall cardiovascular risk [1]. The decline in the LDL-cholesterol concentration with the use of statins is considered the most effective type of pharmacotherapy. Moreover, atorvastatin (ATV) has been preferred over other satins initially due to its effective hypolipidemic effect. So it is widely used as a positive control to lower elevated lipid levels; in studies the dose of ATV for rats ranges from 2.08 to 20 mg/kg/day, but the most commonly used effective dose is 10 mg/kg/day [4–6]. However, statins cannot be applied to all populations worldwide due to side effects, whereas in certain cases their efficacy in lowering cholesterol level is not adequate [7]. About 20% of patients treated with this medication suffer from statin related myalgia, with long-term therapy; it may extends from mild myopathy to fatal rhabdomyolysis. Another impediment with statin therapy is hepatotoxicity, characterized by an elevation of serum transaminases. It has been observed in 1–3% of the individuals treated with atorvastatin and it is predicted that prolonged use of atorvastatin at high doses may lead to rare events of autoimmune hepatitis and deaths [8–10]. Consequently, alternative treatments for lipid metabolism disorders are required.

According to traditional Chinese medicine (TCM), lipid metabolism disorder belongs to the category of phlegm dampness which is formed by the poor transportation of nutrients [11–13]. Erchen decoction (ECD) is a fundamental TCM prescription for the treatment of drying dampness and resolving phlegm [14]. The major components of each herbal medicine in ECD are known to be bioactive components, including flavonoids (e.g., liquiritin, liquiritigenin, hesperidin, rutin, naringin, neohesperidin, and poncirin), triterpenoids (e.g., glycyrrhizin, pachymic acid, and eburicoic acid), and phenolic acids (e.g., homogentisic acid) [15]. With regard to the clinical application of TCM, ECD is extensively used for the treatment of a variety of diseases caused by retention of phlegm dampness, such as obesity, fatty liver, diabetes, and hypertension [16]. Previous studies conducted by our research group and other research groups [17–20] have shown that ECD could improve lipid metabolic disorder. In addition, ECD can regulate glucose and lipid metabolism, reduce lipid disposition, alleviate insulin resistance, and suppress inflammation [21, 22]. ECD, as a Chinese herbal medicine, apply a multicomponent and multitarget approach in the treatment of diseases, so it has definite therapeutic effect with little side effects and wide indications. ECD may be more appropriate for patients with adverse reactions to atorvastatin, as mentioned above. Despite multiple treatment effects observed, the molecular mechanism of ECD in the regulation of metabolic disorder is still unclear.

Lipid metabolism disorder is a direct result of abnormal lipid transport, via lipid transporters and their receptors that play key roles in lipid transport. Caveolae are 50-100 nm stable lipid raft regions abundant in vascular endothelial cells, adipocytes, and fibroblasts [23]. These organelles play key roles in endocytosis and cholesterol homeostasis and are composed of a cholesterol and sphingolipid rich environment that binds to certain receptors, such as gp130 [24–26]. Caveolin-1-3 proteins are responsible for the formation and function of caveolae. Caveolin-1(CAV-1) has been reported to correlate well with lipid metabolism [27]. Caveolae mediate transmembrane lipid transportation and the endocytosis and transcytosis of lipoprotein molecules [28, 29]. Caveolae in the plasma membrane may invaginate and form caveolar vesicles that contain lipids or lipoproteins. With the exception of vesicle-mediated lipid transportation, various receptors in caveolae, such as LDLR, SR-B1, and ABC-A1, mediate lipid efflux [27]. CAV-1 is enriched in caveolae, endoplasmic reticulum, and Golgi bodies and shuttles between the cytoplasm and the cell membrane via the intracellular trafficking of caveolar vesicles [30, 31]. The lipoprotein receptors, such as the very low density lipoprotein receptor (VLDLR) and the low density lipoprotein receptor (LDLR), and the lipid transporters, such as the ATP-binding cassette transporterA1 (ABCA1) and the scavenger receptor class B type I (SRB1) comprise a transmembrane lipid transport system [32–35]. Lipoproteins, including very low density lipoprotein (VLDL), low density lipoprotein (LDL), high density lipoprotein (HDL), and the protein cholesteryl ester transfer protein (CETP) constitute an extracellular lipid trafficking system [36]. The modulation of the levels of lipoprotein receptors by lipid lowering drugs is a mechanism of action that contributes to the decrease of cholesterol and triglycerides in the serum. Despite the potential clinical benefit of ECD in preventing high-fat diet-induced metabolic disorders, the exact mode of action remains poorly defined. In the present study, we aimed to explore the underlying mechanism of ECD lipid lowering action by investigating its effect on the lipid transport system, as determined in caveolae.

## 2. Materials and Methods

### 2.1. Diet and Drug Preparation

The high-fat diet (HFD) contained 60% (cal) fat, 20% (cal) carbohydrates, and 20% (cal) protein with total calorific value of 5.24 kcal/g (D12492, Research Diets, New Brunswick, NJ, USA). The normal diet (NFD) contained 12.1% (cal) fat, 22.5% (cal) protein, and 65.4% (cal) carbohydrate with total calorific value of 3.42 kcal/g (Beijing Huafukang Bioscience Co. Inc., Beijing, China).

According to the textbook of The Taiping Huimin Heji Jufang, Erchen decoction (ECD) comprises four Chinese herbs: Rhizoma Pinelliae (15g), Pericarpium Citri Reticulatae (15g), Poria (9g), and Radix Glycyrrhizae (4.5g). These herbs were purchased from Guoyi Hospital that was affiliated to Fujian University of TCM. ECD was prepared according to the conventional TCM decocting method [1]. All herbs were placed in a cooking pot (porcelain) with 500mL of water and were soaked for 30 min. Subsequently, the herbs were boiled and simmered for 20 min. The liquid was transferred by filtration and water was added to boil the remaining herbs. The process was repeated one more time and the two preparations were mixed to produce a total volume of 250 mL in a glass jar. The mixture was concentrated to 100mL by a rotary evaporator. The doses of ECD we used in the current study were approximately 6 times higher than the standard dose used in clinical practice, according to the dose-equivalence equation between rats and humans [37]. ECD (4.35g/kg) and atorvastatin (ATV, 10 mg/kg) were administered at a dose of 10 mL/kg/d (pure solution).

### 2.2. Animals and Experimental Design

Adult male Sprague Dawley rats weighing 180-200 g were obtained from Shanghai Slac Laboratory Animal Company (Shanghai, China). All rats had free access to water and food under standard laboratory conditions (temperature of 24 ± 2°C; humidity of 55±10%; 12-hour light/dark cycle, light on at 08:00). The experimental protocol was approved by the Fujian University of TCM Ethics Committee for the use of experimental animals (SCXK 2014-004). Following one week of acclimatization (0w), the rats were randomly divided into four groups: NFD (n=14), HFD (n=14), ECD (HFD-fed rats treated with Erchen decoction, n=14), and ATV (HFD-fed rats treated with atorvastatin, n=14). The rats received a HFD for 8 weeks (1-8w) prior the administration of ECD and/or ATV in order to establish a lipid metabolism disorder model. During this period, the success of model establishment was assessed by the measurement of the change in blood lipid levels [38–40]. ECD and ATV were administered to the corresponding animal groups, whereas saline was administered to the NFD and HFD groups by gastric gavage for a period of 4 weeks (9-12w). During the compound administration, HFD-fed rats maintained a high-fat diet in order to prevent self-healing. All rats could eat and drink ad libitum. Food and water consumption were recorded daily, whereas body weight and length were measured weekly. Estimation of energy intake was done indirectly by using the formula: Energy intake = food consumption×food total calorific value (5.24 kcal/g or 3.42 kcal/g). Following 4 weeks of drug treatment, the rats were anesthetized with pentobarbital (40mg/kg body weight) and blood was collected via the abdominal aorta. Their tissues were removed, weighted, and stored at -80°C.

### 2.3. Serum Lipids Content Measurements

Blood was collected from the abdominal aorta into a tube and was centrifuged at 3,500 rpm for 15 min at 4°C. The serum was transferred into a separate vial and stored at 4°C. The concentration of serum triglyceride (TG), total cholesterol (TC), high density lipoprotein cholesterol (HDL-C), low density lipoprotein cholesterol (LDL-C), and free fatty acids (FFAs) were measured using biochemical assay kit according to the manufacturer's instruction (Nanjing Jiancheng Bioengineering Institute, Nanjing, China).

### 2.4. Serum Lipid Transporters Assay

Serum lipid transporters, VLDL, LDL, oxLDL, HDL, and CETP, were measured using the enzyme linked immunosorbent assay kit according to the manufacturer's instruction (Shanghai Meilian Biological Technology Co., Ltd., Shanghai, China).

### 2.5. Real-Time PCR

Total RNA was extracted from liver tissues and visceral fat using RNAiso plus (TaKaRa, Otsu, Japan). Complementary DNA (cDNA) was synthesized using random primers and reverse transcriptase as recommended by the manufacturer instructions (TaKaRa, Otsu, Japan). To evaluate the mRNA expression of lipid transport factors that were derived from caveolae in liver tissues and visceral fat, real-time PCR was conducted using a SYBR Green PCR master mix (TaKaRa, Otsu, Japan) according to the manufacturer's instructions. The reactions were carried out in the Mastercycler ep realplex 4S real-time PCR system (Eppendorf, Hamburg, Germany). The sequences of the primers are described in Table 1. The cDNA was denatured at 95°C for 30s followed by 40 cycles of PCR that included a denaturation step at 95°C for 5 s and an annealing step at 60°C for 30 s. The mRNA levels of the target genes were normalized to the mRNA levels of the *β*-actin gene and the results were expressed as fold changes of the threshold cycle (Ct) value relative to the control samples using the 2^−ΔΔCt^ method [41].

### 2.6. Western Blotting

The liver tissue and visceral fat of each group were homogenized in liquid nitrogen, and whole-cell protein was extracted using lysate buffer that contained proteinase inhibitors (MDL biotech Co., Ltd., Beijing, China). The protein concentration was quantified spectrophotometrically using the BSA protein assay kit (MDL biotech Co., Ltd., Beijing, China). Equal protein amounts were separated by sodium dodecyl sulfate polyacrylamide gel electrophoresis and transferred on polyvinylidene difluoride membranes, which were subsequently blocked with 1% bovine serum albumin and incubated with primary antibodies against CAV-1 (1:500, Cell Signaling Technology, Beverly, MA, USA), VLDLR (1:200, Abcam, Cambridge, UK), LDLR (1:200, Abcam, Cambridge, UK), SR-B1 (1:200, Abcam, Cambridge, UK), ABCA1 (1:200, Abcam, Cambridge, UK), and *β*-actin (MDL biotech Co., Ltd., Beijing, China) overnight at 4°C. Following washing of the membranes with PBS-Tween, the blots were incubated with HRP-conjugated secondary antibodies (1:3,000, Goat Anti-Mouse/Rabbit IgG; MDL biotech Co., Ltd., Beijing, China) for 60 min at room temperature. The protein bands were detected using enhanced chemiluminescence (ECL; MDL biotech Co., Ltd., Beijing, China) and finally scanned by the ChemiDoc MP system (Bio-Rad, USA). The data were quantified by densitometry and presented as the ratio of the target protein levels to the levels of *β*-actin.

### 2.7. Statistical Analysis

Data analyses were performed using the statistical program SPSS 19.0 (SPSS Inc., Chicago, IL, USA). All data were expressed as mean ± SE. Significant differences among groups were evaluated using one-way ANOVA followed by the Tukey tests for post hoc, where equal variances were assumed and/or followed by Tamhane's T2 tests where equal variances were not assumed. The values for the food, water and energy intake, body weight, BMI, and serum lipid were compared by repeated measures ANOVA with a 4×4 (group×time). If the results of the RMANOVA were significant, paired t-tests using a Bonferroni adjustment were conducted as a post hoc analysis. The differences were considered significant for a p value less than 0.05 (*p*< 0.05) and nonsignificant for a p value higher than 0.05 (*p*> 0.05).

## 3. Results

### 3.1. ECD and ATV Do Not Affect Food, Water, and Energy Intake in HFD Rats

**Figure 1:**
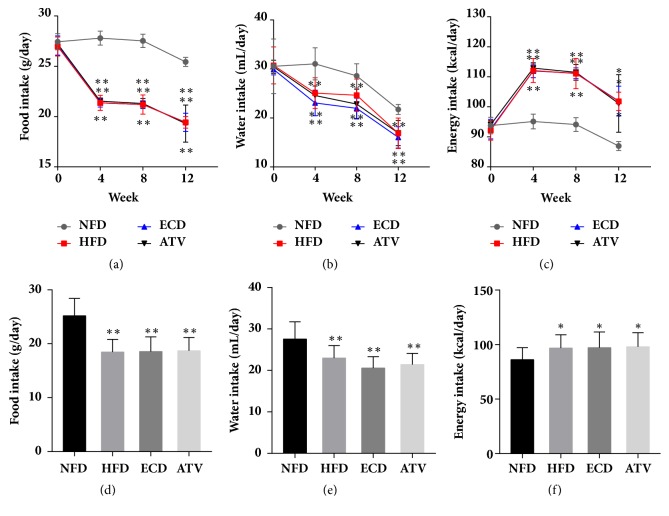
Measurements of daily food, water, and energy intake in the NFD, HFD, ECD, and ATV groups. (a) Food intake, (b) water intake, and (c) energy intake (weeks 0-4-8-12). (d) Food intake, (e) water intake, and (f) energy intake (week 12). The data are presented as mean±SEM (n=14), *∗p*<0.05 and *∗∗p*<0.01 versus NFD.

Before HFD, the parameters daily food, water, and energy intake were not different among the 4 groups of animals, namely, NFD, HFD, ECD, and ATV. Food and water intake were significantly decreased in HFD, ECD, and ATV groups compared with the NFD group following feeding the HFD for four weeks; the effects were maintained until the 12^th^ week (Figures 1(a), 1(b), 1(d), and 1(e)). The opposite effect was noted for the parameter energy intake with regard to the comparison of the 3 groups with the NFD group (Figures 1(c) and 1(f)). No significant differences were observed among the 3 groups with regard to the aforementioned parameters (Figure 1).

### 3.2. The Effects of ECD on the Body Weight, Body Mass Index and Weight of Liver, Perirenal Fat, and Epididymis Fat Tissues

**Figure 2:**
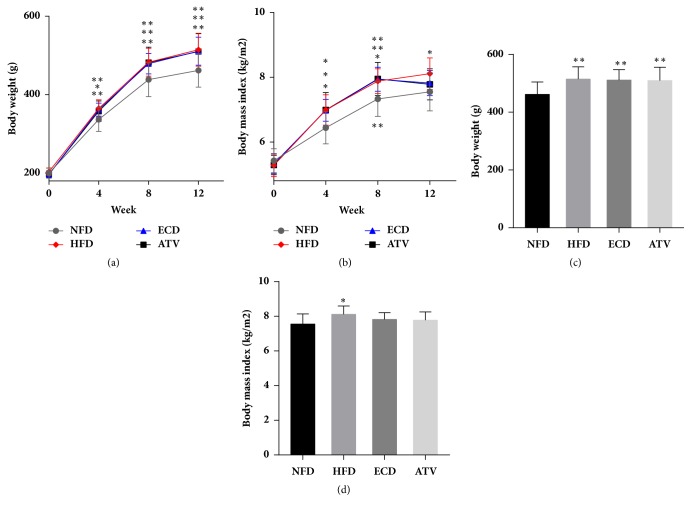
The changes of body weight and Body Mass Index in the NFD, HFD, ECD, and ATV groups. (a) Body weight and (b) Body Mass Index (weeks 0-4-8-12). (c) Body weight and (d) Body Mass Index (week 12). The data are presented as mean± SEM (n=14), *∗p*<0.05 and *∗∗p*<0.01 versus NFD.

On the first day of experiment, the body weight and body mass index (BMI) in each group were not significantly different. Following feeding of the rats with HFD for four weeks, the animals of the groups HFD, ECD, and ATV exhibited significantly higher body weights and body mass indices (BMI) compared with the NFD group (Figures 2(a) and 2(b)). The effects were maintained until the 8^th^ week. After the medication intervention, the body weight in the groups ECD and ATV was still markedly higher than in the NFD group, while the BMI were not significantly different with the NFD group (Figure 2).

**Figure 3:**
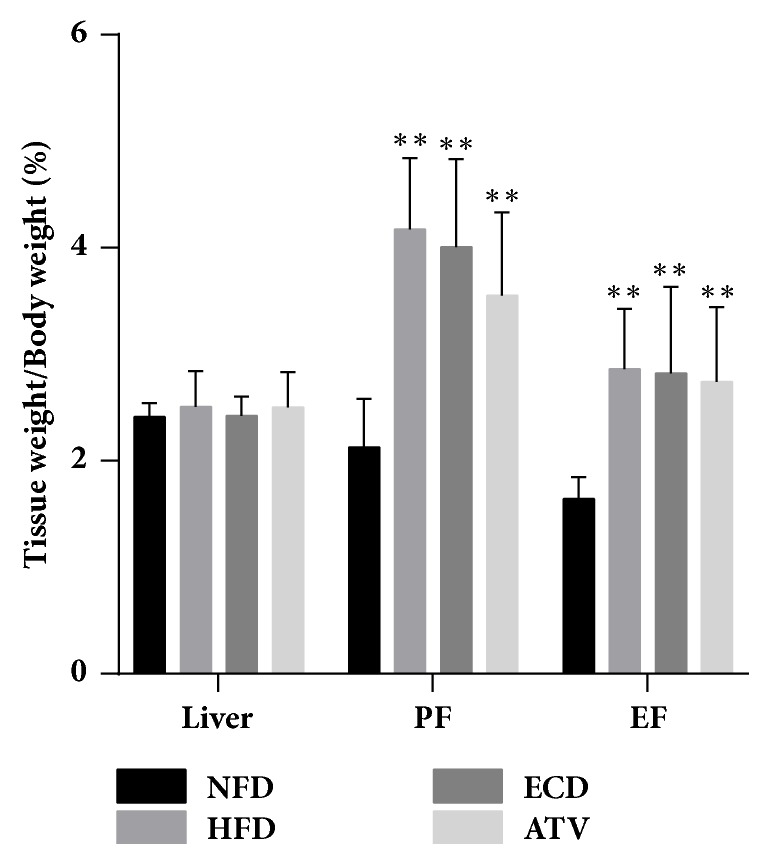
Measurements of percentage of liver, perirenal fat (PF), and epididymis fat (EF) tissue weight to body weight in the NFD, HFD, ECD, and ATV groups (week 12). The data are presented as mean±SEM (n=14), *∗p*<0.05 and *∗∗p*<0.01 versus NFD.

In addition to the body mass index and animal weight, the weights of the liver, perirenal fat, and epididymis fat tissues were measured on week 12. Compared with the NFD group, the percentage of perirenal fat (PF) and epididymis fat (EF) tissue weight to body weight was considerably increased in the groups HDF, ECD, and ATV. However, with regard to the liver tissue, no significant differences were noted for the parameter tissue weight/body weight among the 4 groups (Figure 3).

### 3.3. ECD Affects the Levels of TG, TC, HDL-C, LDL-C, and FFAs in HFD Rats

**Figure 4:**
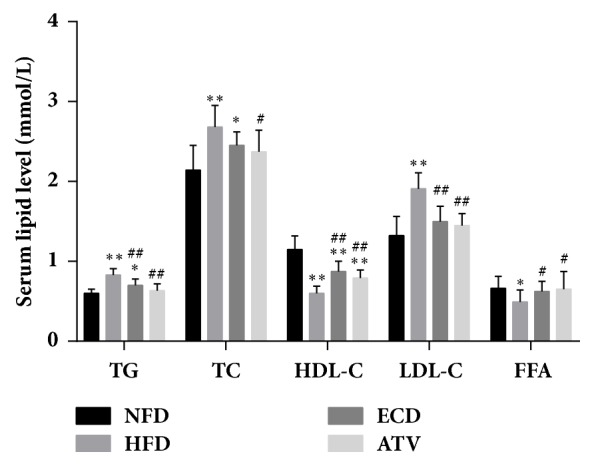
Biochemical measurements of serum lipid levels (TG, TC, HDL-C, LDL-C, and FFA) in the NFD, HFD, ECD, and ATV groups (week 12). The data are presented as mean±SEM (n=14), *∗p*<0.05 and *∗∗p*<0.01 versus NFD; #*p<*0.05 and ##*p<*0.01 versus HFD.

The lipid markers sera TG, TC, HDL-C, LDL-C, and FFAs were measured in the 4 groups (Figure 4). Sera TG, TC, and LDL-C were significantly increased in the HFD group compared with the NFD group, while HDL-C and FFAs were decreased significantly (Figure 4). ECD and ATV supplementation significantly alleviated the HFD-induced disorder in the markers TG, TC, HDL-C, LDL-C, and FFAs(Figure 4). ECD and ATV decreased significantly the TG and LDL-C levels, while they increased significantly the HDL-C and FFAs levels in animals fed HFD (Figure 4). The parameter TC was only significantly decreased following administration of ATV and not ECD compared with the HFD group (Figure 4).

In order to determine the success of model establishment, the change of blood lipid levels was measured and compared before ECD and ATV intervention. Significant elevations were noted for the indices TC during the 8^th^ week and not the 4^th^ week for the groups HFD, ECD, and ATV, whereas HDL-C was significantly decreased in the aforementioned groups on the 8^th^ week (Supplementary Figure 1). The parameters TG and LDL-C were significantly increased on both the 4^th^ and the 8^th^ weeks for the groups HFD, ECD, and ATV (Supplementary Figure 1).

### 3.4. ECD Decreases the Levels of LDL and oxLDL and Increases the Levels of HDL and CETP in HFD Rats

**Figure 5:**
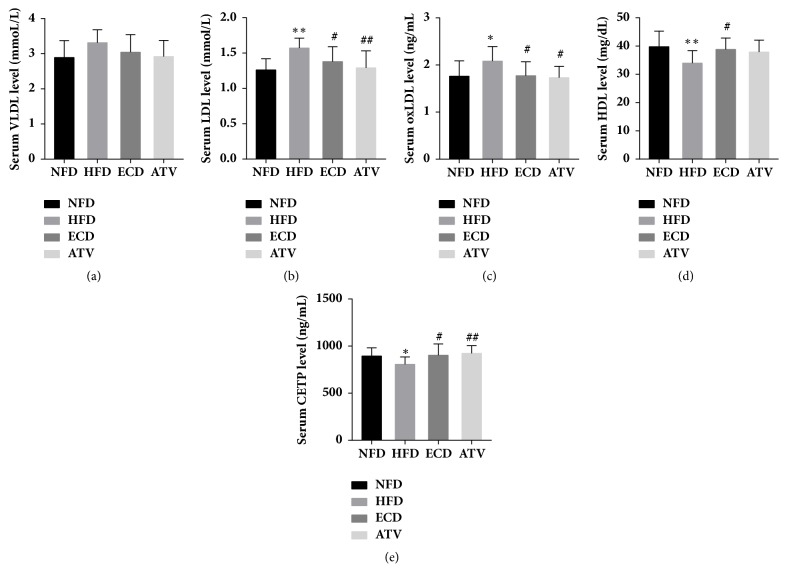
Measurement of the levels of lipoproteins and the cholesterol transfer protein CETP in the NFD, HFD, ECD, and ATV groups (week 12). (a) VLDL. (b)LDL. (c) oxLDL. (d) HDL. (e) CETP. The data are presented as mean±SEM (n=14), *∗p*<0.05 and *∗∗p*<0.01 versus NFD; #*p<*0.05 and ##*p<*0.01 versus HFD.

The serum concentrations of the lipoproteins HDL, VLDL, LDL, and oxLDL as well as the protein CETP were measured in the 4 groups examined. The HFD group exhibited significantly higher levels of LDL and oxLDL and significantly lower levels of HDL and CETP compared with these of the NFD group (Figures 5(a)–5(e)). Following intervention of HFD rats with ECD and ATV, the levels of LDL and oxLDL were significantly decreased, whereas the level of CETP was significantly increased (Figures 5(a)–5(e)). The level of HDL was only significantly increased following administration of ECD and not ATV compared with the HFD group. The level of VLDL did not significantly change in the 4 groups (Figure 5(a)).

### 3.5. The Effects of ECD on the Expression of Lipid Transport Factors in Liver and Visceral Fat of HFD Rats

**Figure 6:**
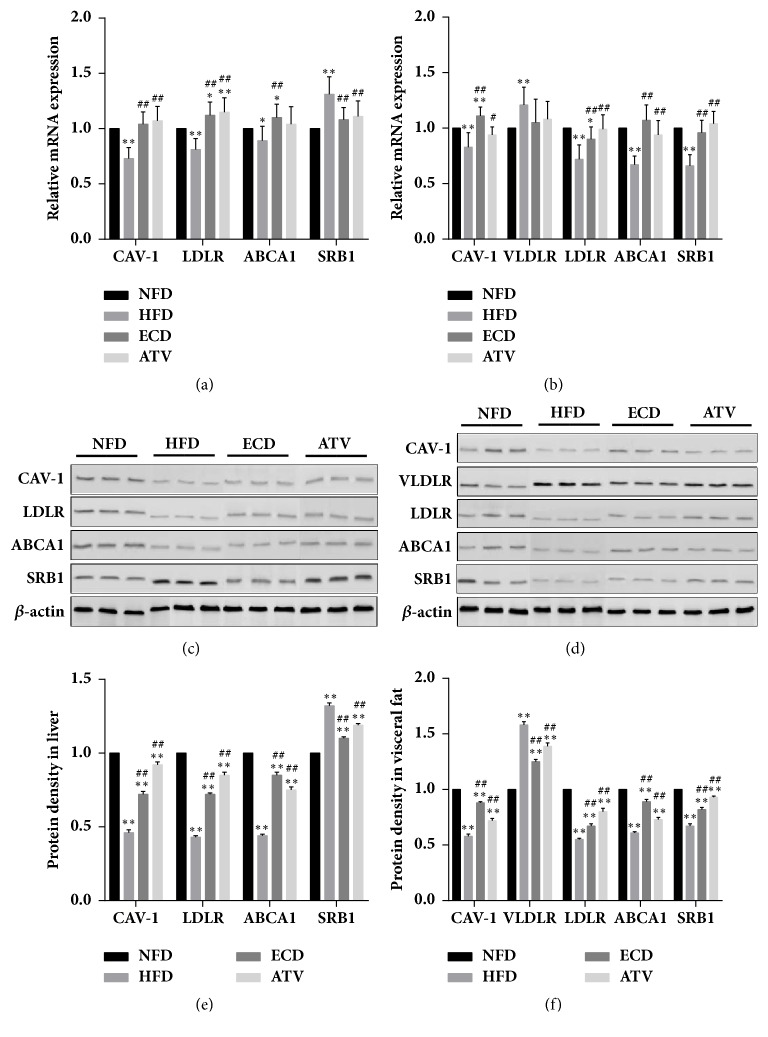
The mRNA and protein expression levels of lipid transporters, as determined by qPCR and Western immunoblotting: CAV-1, VLDLR, LDLR, ABCA1, and SRB1. (a) Liver tissue (mRNA); (b) visceral adipose tissue (mRNA). Western blotting of the expression levels of the proteins CAV-1, VLDLR, LDLR, ABCA1, and SRB1. (c) Liver tissue; (d) visceral adipose tissue. Semiquantification of the expression levels of the proteins examined with regard to the levels of *β*-actin. (e) Liver tissue. (f) Visceral adipose tissue. The data are presented as mean±SEM (n=14), *∗p*<0.05 and *∗∗p*<0.01 versus NFD; #*p<*0.05 and ##*p<*0.01 versus HFD.

To explore the underlying mechanisms of the regulation of ECD on the lipid metabolism disorder induced by HFD, the mRNA and protein expression levels of the protein CAV-1, the receptors VLDLR, LDLR, and the transporters ABCA1 and SRB1 were investigated in the liver and visceral adipose tissues. In the liver, CAV-1, LDLR, and ABCA1 mRNA expression levels were decreased significantly following HFD, while SRB1 mRNA expression levels were markedly increased (Figure 6(a)). ECD and ATV treatment increased significantly the mRNA expression levels of CAV-1, LDLR, and ABCA1, whereas it significantly lowered the mRNA levels of SRB1 in HFD rats (Figure 6(a)). With regard to visceral fat, a similar pattern was noted for CAV-1, LDLR, and ABCA1 mRNA levels (Figure 6(b)). The mRNA levels of VLDLR were significantly increased in the HFD group compared with the NFD group and subsequently decreased in the ECD and ATV groups, although this change was not statistically significant (Figure 6(b)). SRB1 levels in visceral fat tissues were decreased significantly in the HFD rats and subsequently significantly increased in the ECD and ATV groups (Figure 6(b)). These results were confirmed by western blotting (Figures 6(c)–6(f)).

## 4. Discussion

According to TCM, the disorder of lipid metabolism is considered to be affected by the retention of phlegm dampness. HFD causes spleen and stomach impairment and leads to the accumulation of grease in the body, and finally the excess grease induces the retention of phlegm dampness [10]. The present study examined the lipid lowering effects of ECD in rats that were fed a high-fat diet. ECD was capable of reducing the levels of TC, TG, and LDL-C indices and further responsible for the increase in the levels of HDL-C in HFD rats, while it concomitantly did not affect considerably the food, water, and energy intake of HFD rats. These effects were comparable to the antiatherogenic agent atorvastatin (ATV). Additional investigation in the mechanism of action of the lipid lowering effect of ECD revealed that this extract could upregulate CAV-1, LDLR, and ABCA1 levels in the liver and visceral fat tissues and downregulate SRB1 levels in the liver while upregulate SRB1 levels in the visceral fat tissues of HFD animals. Taken collectively, the data suggested that ECD was an effective lipid lowering mixture of compounds that acted by regulating the apolipoprotein, apolipoprotein receptor, and lipid transport levels.

A limited number of studies have been conducted with regard to ECD and its applications for the treatment of various disorders. ECD has been reported to be used therapeutically for chronic bronchitis [42]. Furthermore, ECD ameliorated nonalcoholic steatohepatitis in a rat model of high-fat diet, as determined by reduced hepatic lipid disposition and liver injury and lower serum biochemistry markers, whereas in a similar study it was shown to improve insulin resistance and liver damage in rats, as evidenced by serum aminotransferase levels and histopathological examination [22, 43]. ECD is a basic herb for the treatment of drying dampness and resolving phlegm and has been widely reported to improve the disorder of lipid metabolism [44, 45]. Animal experiments indicated that ECD could reduce blood glucose and regulate lipid metabolism [46], so as to ameliorate insulin resistance [23] or atherosclerosis [47]. In the present study, treatment with ECD significantly reduced the levels of TC, TG, and LDL-C and improved the levels of HDL-C and FFA in HFD rats. Furthermore, following HFD, ECD intervention decreased the levels of LDL and oxLDL and increased the levels of HDL and CETP, suggesting that ECD can effectively ameliorate disturbance of lipid metabolism induced by HFD in rats. A previous study conducted by Zhang and coworkers reported similar findings, and ECD was shown to improve TC and LDL-C levels of early hyperlipidemia and atherosclerosis animal models [48]. These changes were evident on weeks 2 and 4 of the HFD animals [48]. The present study is in agreement with the aforementioned changes in the lipid parameters and the effects of ECD, although there were some differences between the studies, such as the animal model (lipid metabolism disorder model versus hyperlipidemia and atherosclerosis model), the dose (4.35g/kg versus 4.8g/kg), and the time point (8 weeks versus 5 days after duplicated the model) of administration.

**Figure 7:**
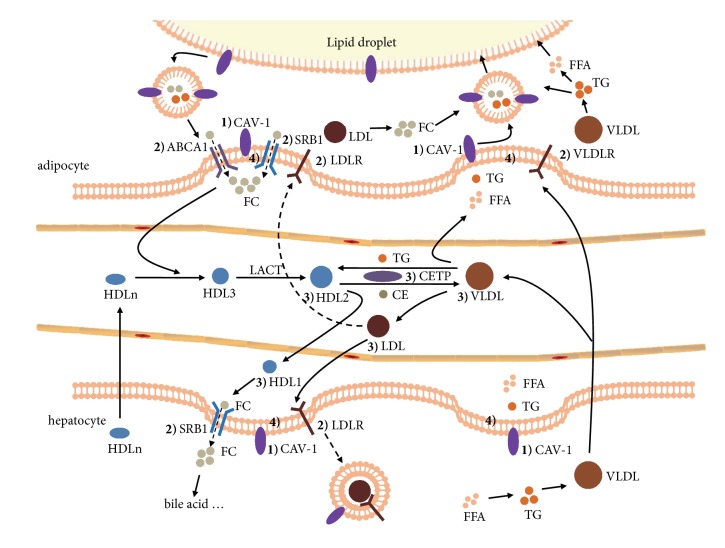
The model of lipid transport in caveolae. This model includes (1) an intracellular lipid trafficking system of CAV-1, (2) a transmembrane lipid transport system of lipoprotein receptors, (3) an extracellular lipid trafficking system of lipid transporters, and (4) a caveolae transport center.

Based on the data presented in the current study, we hypothesized that the regulation of lipid transport was carried out in caveolae (Figure 7). These structures are considered the underlying factors required for ECD to alleviate dry dampness and resolved phlegm and ultimately to regulate lipid metabolism disorders. Caveolae is a site where lipids are transported intracellularly and extracellularly. CAV-1 is considered an intracellular lipid trafficking factor that can transport lipids and shuttles between the cytoplasm and the cell membrane via the caveolar vesicles [49, 50]. Cholesterol efflux is increased in HepG2 cells transfected with a CAV-1 expression plasmid DNA [51]. Moreover, it was confirmed that CAV-1 is a lipid-droplet protein [52–54] that can transfer lipids to lipid droplets for storage. In addition, VLDLR and LDLR can mediate the necessary lipoprotein/lipid transport into the cells, so as to avoid the accumulation of lipids in the blood [55–58], whereas ABCA1 can mediate cholesterol efflux. ABCA1 in adipocytes influences adipocyte lipid metabolism, body weight, and whole-body glucose homeostasis [59]. It has been shown that the expression of ABCA1 in macrophages from CAV-1 knockout mice is lower than that of the control group, indicating that CAV-1 is involved in regulating ABCA1-mediated cholesterol efflux [60]. HDL presents or accepts cholesterol while anchored to plasma membranes via its receptor, SRB1. SRB1 mediates not only CE selective uptake (mainly in hepatocytes) but also cholesterol efflux (mainly in perithelial cells) [30]. It has been reported that caveolae present in cell membranes are the initial receiving sites of SRB1-mediated CE intake and 80% of CE accumulates in caveolae [61]. LDLR, SRB1 and ABCA1 are localized in caveolae [62].

To confirm this hypothesis, we measured the expression levels of the genes involved in the lipid transport in the rat liver and visceral fat tissues. HFD decreased the expression levels of CAV-1, LDLR, and ABCA1 and increased the expression levels of SRB1 in the liver tissues. Concomitantly, the expression levels of CAV-1, LDLR, ABCA1, and SRB1 were inhibited by HFD in the visceral fat, while the expression of VLDLR was increased. Following ECD intervention, the abnormal expression levels of these genes were reversed to their initial baseline values, indicating that the effect of ECD in the improvement of the disorder of lipid metabolism was mainly attributed to its ability to regulate the expression levels of lipid transporters in caveolae.

The mechanism by which ECD exerts lipid lowering effects in HFD animals remains unclear. Previous studies have shown that ECD could downregulate the MAPK JNK and the transcription factor AP-1, while it could further upregulate p-PKB in HFD rats that had nonalcoholic steatohepatosis [22]. The study by Zhang et al. further demonstrated that ECD could decrease TNF-*α* and NF-*κ*Β levels in HFD-induced hyperlipidemic rats [43]. These studies have examined the mechanism of action of ECD with regard to cell signaling and inflammation, respectively. However the modulation of the expression levels of the lipid transporters CAV-1, LDLR, ABCA1, and SRB1 by ECD in the liver and visceral fat tissues of HFD animals has not been investigated to date. However, previous studies have shown that ATV treatment in rainbow trout,* in vitro* hepatocytes, and hyperlipidemic men can increase the levels of LDLR [63–65]. Since ECD exhibited similar LDL-C and TC lowering effects, to those of ATV it is conceivable that a similar pattern of regulation with regard to the protein CAV-1 and the receptor LDLR would be expected, as these markers are both involved in cholesterol metabolism. More importantly, ATV was reported to increase the levels of ABCA1 under HFD conditions in the liver and adipose tissues of hypertensive rats, which agrees with the present findings [66]. The present study adds further insight to the mechanism of lipid lowering effects mediated by ECD.

Long-term intake of high-fat diet induces a variety of reactions to cause lipid metabolism disorder in the body, such as insulin resistance (IR) [22, 43], inflammation [67], and endoplasmic reticulum (ER) stress [68]. These reactions will promote abnormal fat synthesis and decomposition through related factors and signaling pathways. Considering the key role of IR activation in regulating lipid metabolism, early studies have shown that ECD can regulate lipid metabolism by improving IR. Much data demonstrate a fundamental involvement of caveolae in insulin action [69]; this is why we studied the mechanism of ECD on regulating lipid metabolism from the perspective of caveolae. Now we are also exploring the effect of ECD on ER stress caused lipid metabolic disorders, and we will continue to investigate the molecular mechanisms and active constituents of ECD on regulating lipid metabolism in the future.

## 5. Conclusions

In summary, we have demonstrated that ECD exhibits a similar therapeutic effect on lipid metabolism disorder with that of ATV in HFD rats. Treatments with ECD ameliorated the lipid transport in caveolae. Further studies should be conducted to identify the effective constituents of ECD and molecular targets that are responsible for ameliorating the lipid metabolism disorder by ECD.

## Figures and Tables

**Table 1:** Primer sequences used for real-time PCR analysis.

Gene	Forward primer	Reverse primer
CAV-1	TAAACCCATTCCTGCTCTCC	CGCCTCCCAGTCTTCCTATT
VLDLR	CACCAAAGTATGTGACCAGGAA	AACAGCCACCGTTATTGACC
LDLR	TAGGGTTTCTGCTCTTCACCA	GCCACCACATTCTTCAGGTT
SRB1	GGACAAGCAGTTCCAGATCC	GCCAATTCAGTGTTCAGGTG
ABCA1	TGCTTCCGTTATCCAACTCC	TCCCTAATGCTGGTGTCCTT
*β*-Actin	CACCCGCGAGTACAACCTTC	CCCATACCCACCATCACACC

## Data Availability

The data used to support the findings of this study are included within the article.

## Supplementary Materials

Supplementary Materials Supplementary Figure 1: biochemical measurements of serum lipid levels in the NFD, HFD, ECD, and ATV groups. (A) TG, (B) TC, (C) HDL-C, and (D) LDL-C (weeks 0-4-8). The data are presented as mean± SEM (n=14); *∗*p<0.05 and *∗∗*p<0.01 versus NFD.
