# Supplementary material for: Erchen Decoction Ameliorates Lipid Metabolism by the Regulation of the Protein CAV-1 and the Receptors VLDLR, LDLR, ABCA1, and SRB1 in a High-Fat Diet Rat Model
Source: Evid Based Complement Alternat Med. 2018 Oct 8;2018:5309490. doi: 10.1155/2018/5309490 (PMC6196931; doi:10.1155/2018/5309490)

Supplementary Figure1: Biochemical measurements of serum lipid levels in the NFD, HFD, ECD and ATV groups. (A) TG, (B) TC, (C) HDL-C, and (D) LDL-C (weeks 0-4-8). The data are presented as mean± SEM (n=14), **p*<0.05, ***p*<0.01 vs. NFD.

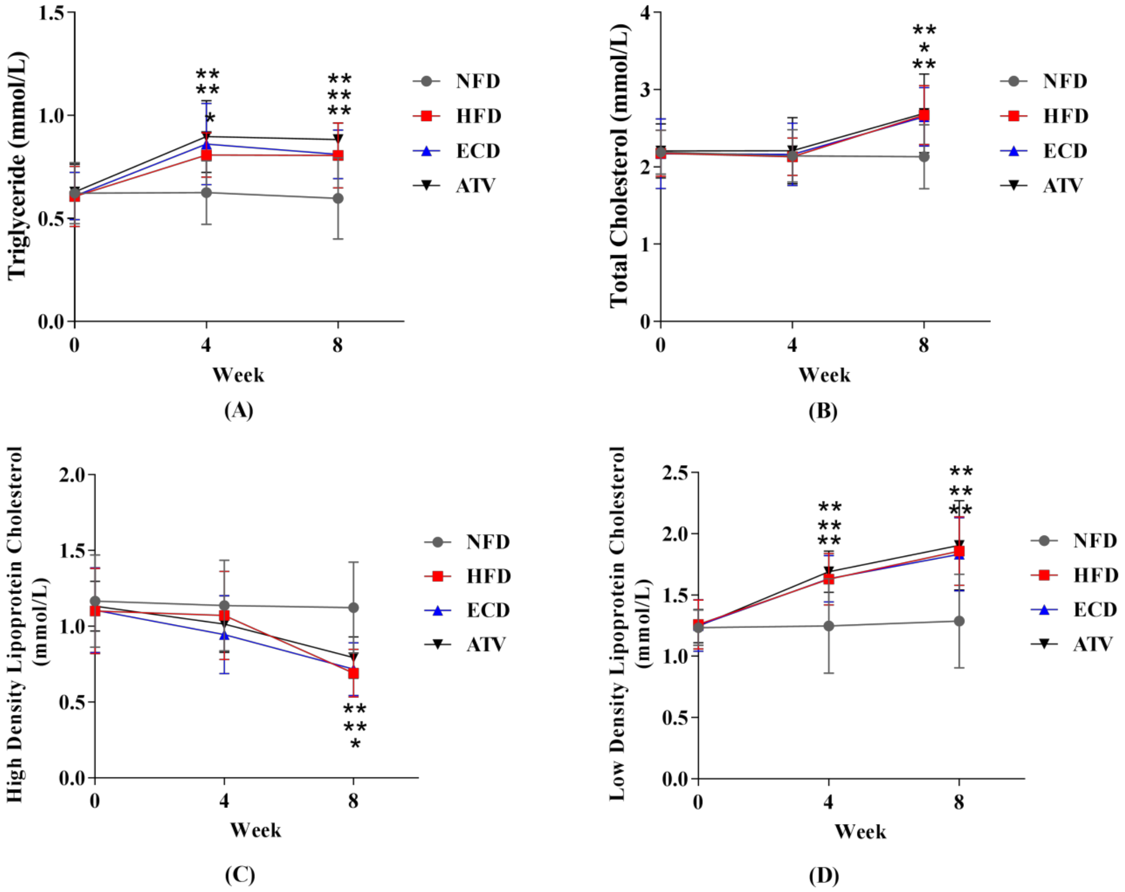

Supplement: Supplementary Materials — Supplementary Figure 1: biochemical measurements of serum lipid levels in the NFD, HFD, ECD, and ATV groups. (A) TG, (B) TC, (C) HDL-C, and (D) LDL-C (weeks 0-4-8). The data are presented as mean± SEM (n=14); ∗p<0.05 and ∗∗p<0.01 versus NFD. [file 5309490.f1.pdf]
